# Eating and nutrition habits in young competitive athletes: a comparison between soccer players and cyclists.

**Published:** 2014-12-19

**Authors:** Giorgio Galanti, Laura Stefani, Irene Scacciati, Gabriele Mascherini, Gabriella Buti, Nicola Maffulli

**Affiliations:** * Sports Medicine Center –University of Florence –Italy; ** Dietetic Unit-University of Florence – Italy; *** Department of Musculoskeletal Disorders, Faculty of Medicine and Surgery, University of Salerno, Salerno, Italy; and Queen Mary University of London, Barts and The London School of Medicine and Dentistry, Institute of Health Sciences Education, Centre for Sports and Exercise, UK

**Keywords:** *nutrition*, *young athletes*, *eating habits*, *soccer players*, *cyclists*

## Abstract

The study evaluated the dietary habits in two groups of young athletes, practicing two different sports: soccer players and cycling. The dietary habits of 47 athletes were investigated by questionnaire. Body Mass Index, Fat Mass, Free Fat Mass, Total Body, Intracellular, Extracellular Water and Phase Angle were measured by bioimpedance. The *t*-Student test for unpaired data was used. Significance was set at P < 0.05. Body Mass Index was similar between the groups, while total body water and extracellular water were significantly higher in the soccer player group (soccer players: 63.8±1.96%; cyclists : 59.8 ± 8.7%; and soccer players 43.9±3.1%, cyclists 43.8 ±2.1%, respectively). Fatty mass of the soccer player group (14.5±2.9%) was significantly lower than that of the cyclist group (19.5±3.6%). Daily food intake was similar between the two groups (2844 kCal/die for soccer players /2630 kcal/die for cyclists), and lower than recommended. There was a low intake of Calcium (soccer players 1120±128.9 mg/die, cyclists 718±309 mg/die) for both groups, and a low intake of Potassium for soccer player (2576 mg/die ± 52.4) The caloric intake of adolescent athletes is lower than recommended. Body composition is significantly different between soccer players and cyclists.

## I. INTRODUCTION

Proper nutrition supports the achievement of optimum athletic performance (1). A balanced diet should ensure adequate caloric, macro- and micro-nutrients intake (1, 2, 3, 4, 5). Especially in adolescent athletes, in addition to the energy requirements arising from exercise, appropriate dietary habits will be carried on to adulthood and in parallel in presence of physical activity, the risk of incorrect lifestyle can be reduced (1).

Specific guidelines have been developed for athletes (6). Despite strong commercial pressure, physically active individuals generally do not need supplements in addition to a normal diet (1, 7, 8, 9). This study has been conducted in adolescent, practicing two popular sports and characterized by a different workload in terms of resistance and aerobic work load and with potential diverse training such as soccer players and cyclists. The present investigation is aimed to evaluate their habits, using detailed medical history, to ascertain the knowledge of nutrition in sport and to verify adhesion to the recommendations of the Italian Nutrition Agency (Reference Levels of Nutrients identified by LARN) (10), and also to compare nutritional habits of these two groups of athletes, assessing any possible differences in energy intake, choice of food, and distribution of food intake. It has been also investigated the adherence to the reference levels of nutrients and energy for the Italian population.

## II. METHODOLOGY

We studied two groups of elite athletes: 17 male cyclists aged between 14 and 16, belonging to four different teams with similar levels of competition and training loads, and 30 young soccer players from Fiorentina Football Club youth team, aged between 15 and 16. All athletes were training regularly and competing in national competitions for their age group. The cyclists trained 11 months a year for an average of 3 hours per day in addition to competing during the racing season. The soccer players trained about 10 months a year, with a frequency of 4 sessions a week with a weekend game.

The investigation was performed during the sports season. All subjects underwent a full medical check up and were questioned extensively regarding food intake. They underwent bioimpedenziometry and answered a questionnaire on nutritional knowledge. None of them assumed nutritional supplements or ergogenic substances.

### Food habits analysis

All the young athletes were invited to answer questions about the type of food, the portions and frequency of consumption of various foods and beverages, and the possible use of dietary supplements. We used a dedicated software (Winfood, Medimatica Srl; Colonella (Te)-Italy) to calculate the daily intake of macronutrients and micronutrients. The quantification of food intake was undertaken using pictures of food depicted in different portions with known weights from a dedicated photographic archive. The software allowed to identify the following parameters:

- Percentages of total energy intake at breakfast, morning snack, lunch, afternoon snack, dinner and evening.
- Profiles of macronutrients intake and their percentage contribution to the total energy intake
- Profile of protein intake in animal and plant components;
- Total carbohydrate contribution, including individual components (starch and oligosaccharides)

At the end of the first part of the interview, all athletes answered questions to test their knowledge on nutrition and to ascertain where they had received the information. The main questions used to investigate the awareness in the nutrition habits athletes are reported below:

- Do you think that nutrition is important for the maintenance of good health?
- Do you think that nutrition is important for the maintenance of sports performance?
- Did you receive any kind of diet information?

If the athletes answer ‘YES’ to this last question, they were asked to specify from whom they received the information.

Considering that all the athletes investigated were Italians, the LARN parameters were applied.

### Body composition analysis

Body composition was analyzed using bioelectrical impedance to estimate the state of hydration, free fat mass and fat mass. The data were obtained according to the recommendations of the NIH Consensus Statement (11). The measurements were carried out on the right side of the body through the tetra-polar device model 101S RJL system, through the bioelectrical method Vector impedance (12) Akern srl Florence with an alternating electric current of 800 µA at a frequency of 50 kHz. All measurements were taken in a room with temperature of 22–24 °C. The data were processed through a software system to derive estimates of total body water, extracellular water, intracellular water, body cell mass and fat mass, expressed as weight in kilograms and percentage. The results obtained from the two groups were then compared. The values related to the desirable range for the body impedance analysis (13) are reported in Table 1.

### Statistical analysis

The SPSS program (SPSS 17.0 software.) was used for statistical analysis. The *t*-Student’s test for unpaired data was used. A P <0.05 was considered statically significant. The association among the parameters was tested using the *r* correlation coefficient.

## III. RESULTS

### Anthropometric and water distribution parameters

All data are expressed as mean ± SD. No significant differences were found among the general characteristics of the two groups analyzed: the average weight of the cyclists was 4.9 kg less than the soccer players (cyclists 65.7 ± Kg 8.0; soccer player 70.6 ±6.5 Kg). Cyclists were also less tall (cyclists 174.8 ±7.4 cm; soccer players 177.8 ± 6.4 cm). The Body Mass Index was within normal range (14), and no significant differences (soccer players 22.3 ± 0.9Kg*m^−^2; cyclists 21.46 ± 2.0) were found between the two groups of athletes. Bioelectrical impedance analysis data are reported in Table 2. The average value of phase angle derived from the ratio between resistance and reactance, whose normal values range between 6 and 9, is significantly different between the two groups of athletes (soccer players 7.1±0.5; cyclists 7.3±0.6 p <0.05). The total amount of body water, normally greater than 60%, is significantly higher in soccer players than in cyclists (cyclists 63.8 ± 1.9 Lt; soccer players 59.8 ±8.7 Lt). From the analysis of body water distribution divided in intra and extra cellular districts, no difference in extra cellular water (soccer players 43.87 ±3.0 %; cyclists 43.8±2.1), was found but there was a significant disparity in intracellular compartment (soccer players 57.4±1.9%; cyclists 56.1±2), which represents the metabolically active body mass. The average percentage of fat mass of cyclists was significantly greater than in soccer players (cyclists 19.5 ±3.6 %; soccer players 14.5 ±2.9 %). Comparing the data to the values of the main guidelines that suggest 18% as a benchmark, cyclists presented slightly greater mean percentage of body fat (Table 1). On the contrary, only two soccer players exceed 18% fatty mass, while more than half of the cyclists had values in excess of 18%. Fatty free mass was significantly higher in the soccer players (soccer players 85.5 ±2.9%; cyclists 80.5 ±3.6%) with higher values of body cellular mass (%) (58.9 ± 2.2 in soccer players and 56.5 ± 9.0 in cyclists) and of basal metabolism rate (kCal) (1742.6 ± 156.2 for soccer players and 1535.4 ± 193.1 for cyclists)

No evidence of a statistically significant association was found between total body water, intra cellular water and fatty mass in both groups (CI: 0.29 for cyclists and 0.24 for the soccer players).

### Evaluation of the food intake

Caloric intake and consumption of macronutrients in the two groups as well as the recommended percentages are reported in Table 3, while the profile of fat intake and micronutrients such as minerals and vitamins intake are reported in Tables 4. Calcium intake is low in both groups, but especially in the cyclists, whose ingestion is about half of what recommended. Potassium intake is below the LARN in soccer players but not in cyclists (Table 5). Both groups show insufficient daily intake of folic acid and vitamin B6, and extremely high intake of Vitamin C in soccer players (Table 4). The daily energy intake and meal distribution were comparable between the two groups. Most athletes showed a balanced energy intake between main meals and snacks, except for breakfast, which results to be appropriate for the nutrients and with a good choice of foods, but scarce from the point of view of energy intake (Table 5). Comparing the annual distribution of energy components, cyclists consumed large amounts of carbohydrates, especially complex carbohydrates, and proteins; on the other hand, soccer players had a greater fat intake. There were no differences in dietary habits between the two types of sport.

### Questionnaire results

Both groups of athletes reported that maintenance of good health and an acceptable sports performance were considered of principal importance. Cyclists reported that 70% received food advice provided in most cases by parents, doctors or coaches. Only 60% of soccer players stated that they received dietary recommendations, mainly from coaches, team doctors, parents and masseurs; in only one instance the information was provided by a registered dietitian.

## IV. CONCLUSION

Nutritional and eating habits have been of particular interest in sports, especially given their effects on athletic performance (15, 16, 17,30). General recommendations need to be adjusted by sports nutrition experts to accommodate the unique concerns of individual athletes regarding health, sports, nutrient needs, food preferences, and body weight and body composition goals (4,31). Investigations on dietary and nutritional habits play an important role in the analysis of athletes’ lifestyle (18). Questionnaires are commonly used to ascertain the adherence to recommendations.

The present investigation should be considered a pilot study. Nevertheless, the results suggest that teenage athletes tend to adhere to the international guidelines on nutrition (19, 20, 21, 22).

Nutritional issues seem to be of great importance in younger athletes. The average caloric intake of all athletes was slightly lower than the estimated requirements, while the intake of macro and micronutrients was in agreement with the relevant guidelines (13, 24, 25).

In both groups, the amount of vitamins and minerals, with the exception of folic acid was higher than the recommended requirements. The consumption of fruit and vegetables was adequate, and in agreement with the recommendations for the general population (10). Body composition data were different between the two groups of athletes studied, particularly if hydration status is considered Despite the broadly similar food intake and daily meal distribution, the differences could be attributed to the diverse type of sport practiced and therefore to the impact that sports training can exert on anthropometrics variables (26, 27).

These anthropometrics parameters are partially in contrast with the data normally reported in the literature. We however underline that the population investigated was composed of non-professional athletes. In addition they were young athletes, not yet completely mature, and therefore the differences in the body composition can be attributed to this aspect.

Further investigations involving several types of different sports and larger samples will be needed to confirm and extend our knowledge on these preliminary aspects of the relationship between food intake and sport activity. Also, female athletes should be investigated (29). Some other aspects about a possible association between nutrition and cardiovascular performance may be useful, as would be the evaluation of the prevalence and incidence of musculoskeletal injuries in the presence of specific eating habits.
